# Optical Properties and Fluence Distribution in Rabbit Head Tissues at Selected Laser Wavelengths

**DOI:** 10.3390/ma15165696

**Published:** 2022-08-18

**Authors:** Alaa Sabeeh Shanshool, Ekaterina Nikolaevna Lazareva, Omnia Hamdy, Valery Victorovich Tuchin

**Affiliations:** 1 Science Medical Center, Saratov State University, 410012 Saratov, Russia; 2 Laboratory of Laser Molecular Imaging and Machine Learning, Tomsk State University, 634050 Tomsk, Russia; 3 Department of Engineering Applications of Laser, National Institute of Laser Enhanced Sciences (NILES), Cairo University, Giza 12613, Egypt; 4 Laboratory of Laser Diagnostics of Technical and Living Systems, Institute of Precision Mechanics and Control, FRC “Saratov Scientific Centre of the Russian Academy of Sciences”, 410028 Saratov, Russia

**Keywords:** tissue optical properties, optical fluence rate, transcranial laser irradiation, optical transmittance, brain, diffuse reflectance, Kubelka–Munk model, Monte-Carlo simulation, finite-element solution, Inverse adding-doubling (IAD)

## Abstract

The accurate estimation of skin and skull optical properties over a wide wavelength range of laser radiation has great importance in optogenetics and other related applications. In the present work, using the Kubelka–Munk model, finite-element solution of the diffusion equation, inverse adding-doubling (IAD), and Monte-Carlo simulation, we estimated the refractive index, absorption and scattering coefficients, penetration depth, and the optical fluence distribution in rabbit head tissues ex vivo, after dividing the heads into three types of tissues with an average thickness of skin of 1.1 mm, skull of 1 mm, and brain of 3 mm. The total diffuse reflectance and transmittance were measured using a single integrating sphere optical setup for laser radiation of 532, 660, 785, and 980 nm. The calculated optical properties were then applied to the diffusion equation to compute the optical fluence rate distribution at the boundary of the samples using the finite element method. Monte-Carlo simulation was implemented for estimating the optical fluence distribution through a model containing the three tissue layers. The scattering coefficient decreased at longer wavelengths, leading to an increase in optical fluence inside the tissue samples, indicating a higher penetration depth, especially at 980 nm. In general, the obtained results show good agreement with relevant literature.

## 1. Introduction

The use of lasers in biomedical diagnostics and therapy requires spectroscopic measurements of the beams that propagate through the examined tissue or organ. Optical diagnosis methods have a high impact because they are safe, minimally invasive, and non-destructive. In neurology and brain examination, light travels through the scalp, skull, and brain. Despite the important effects of the upper tissue layers on measuring brain hemodynamics, the effect of the scalp and skull on the sensitivity and intensity of laser radiation has not been well characterized. Innovation headways in photonics have made massive advancements toward the development of creative strategies and systems for clinical practical optical imaging, laser surgery procedures, biomedical diagnostics, and phototherapy. The progress of optical biomedical techniques and methods focuses on the investigation of the optical properties of tissues, which characterize the viability of tissue optical testing and light activity on the tissue and, when known (estimated), allow for predicting the exact photon spread directions and the fluence rate distribution inside irradiated tissues [1]. Tissue removal, coagulation, laser cutting, and many other applications depend on the spectroscopic properties of tissues for their accuracy. Therefore, information regarding the optical properties of tissues is essential for the interpretation and evaluation of the diagnostic data and for the prediction of the absorbed light and energy distribution for therapeutic and surgical use. Various publications connected with the assurance of brain tissue optical properties and demonstrating the fluence rate distribution are accessible in the literature [1,2,3,4,5,6,7,8,9,10,11,12,13,14,15,16,17,18,19,20,21,22]. The examination of these publications shows that the optical properties of human and different animal brain tissues have been explored in the visible and NIR spectral ranges. However, these analyses exhibit an assortment of cutting edge biomedical advances that require exact information on the optical properties of the tissues [10,11,12,13,14,15,16].

The optical properties of tissues that assume a significant role in tissue characterization are the absorption coefficients (μ_a_), scattering coefficients (μ_s_), and the reduced scattering coefficients (μ_s_′), including the refractive index (RI) and the anisotropy factor (*g*). These parameters are high-wavelength-dependent and give practical data about the tissue, for example, the hemoglobin content, tissue oxygenation, and water fraction. However, assessing the optical properties of any tissue requires estimations regarding the diffuse reflectance (*R*_d_), diffuse transmittance (*T*_d_), and collimated transmittance (*T*_c_). Experimentally, there are several methods to obtain these estimates, either by integrating spheres or using techniques in light with distant detector arrays or with other devices. Optical differentiation is a useful tool in biomedical diagnosis, for the most part, as a result of its safety. As per histopathology, the values of the tissue optical properties will contrast for different tissues and consequently could be utilized for differentiation of the norm and pathology. The optical fluence rate distribution inside the tissue limits depends on the optical boundaries. Therefore, presenting the parameters of such distributions can give an optical method for biomedical diagnosis [17,18,19,20,21,22,23,24,25,26,27,28,29,30,31]. This paper presents the results of the measurements and estimation of tissue optical properties, where an experimental setup was implemented to measure the diffuse optical reflectance and transmittance of the ex vivo samples of the head skin, skull, and brain at 532, 660, 785, and 980 nm laser wavelengths. Using the measured values, the optical parameters of the samples were determined utilizing an inverse adding-doubling (IAD) method and the Kubelka–Munk model. The assessed values of the optical parameters were utilized in the diffusion equation to simulate the fluence rate at the tissue surface using the finite element method. The results were confirmed with the Monte-Carlo simulation, which included modeling of the in-depth fluence rate distribution for the three-layer head tissue.

## 2. Materials and Methods

### 2.1. Sample Preparation

**Figure 1:**
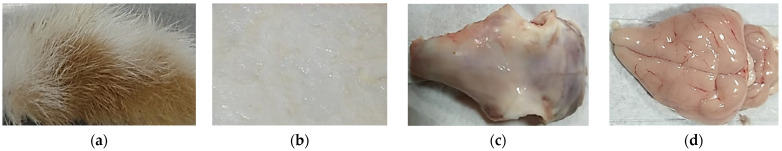
The studied tissue samples: skin before (**a**) and after (**b**) shaving, skull (**c**), and brain (**d**).

Ten adult rabbits were acquired from a local breeder who sold rabbits for consumption. Then, the animals were sacrificed one at a time on different days. All of the animal studies were approved by the Animal Care and Experimentation Committee of the university. Skin (scalp), skull, and brain samples were manually removed from the heads using appropriate scalpels for ex vivo investigation. The average thickness of the skin was 1.1 mm, skull was 1 mm, and brain was 3 mm. For the RI measurements, several samples of rabbit brain tissue were prepared with a 3 mm thickness. For the spectral measurements, 10 samples were prepared with a 0.5 mm thickness. The skin samples were shaved for accurate measurements (see Figure 1b). Transmittance and reflectance and measurements were done in the fresh native state and the rest of the samples were placed in the refrigerator at −20 °C. The studied samples were positioned between two 1 mm thick microscope glass slides for the spectroscopic measurements.

### 2.2. Experimental Arrangements

An optical setup based on an integrating sphere (McPherson, KS, USA) coupled with a commercial digital fiber spectrometer (STDFSM, Touptek Photonics Co., Ltd., Hangzhou, China) was utilized for measuring the tissue transmittance and reflectance. The sphere had two output ports with diameters of 25 and 15 mm, while the diameter of the radiation inlet port was 10 mm. The measurements were obtained using four CW laser sources (PGL-DF, Changchun New Industries Optoelectronics Tech. Co., Ltd., Changchun, China) at wavelengths of 532 nm (~100 mW power, 2 mm beam diameter, near TEM_00_), 660 nm (~130 mW power, 3.5 mm beam diameter, near TEM_00_), 785 nm (~126 mW power, 4 mm beam diameter, near TEM_00_), and 980 nm (~108 mW power, 3 mm beam diameter, multimode) [22]. An analysis of the results was completed using the spectrometer software and Matlab R2018a program.

### 2.3. Tissue Diffuse Light Measurements

**Figure 2:**
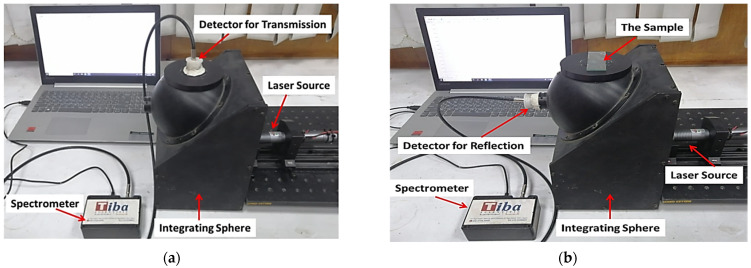

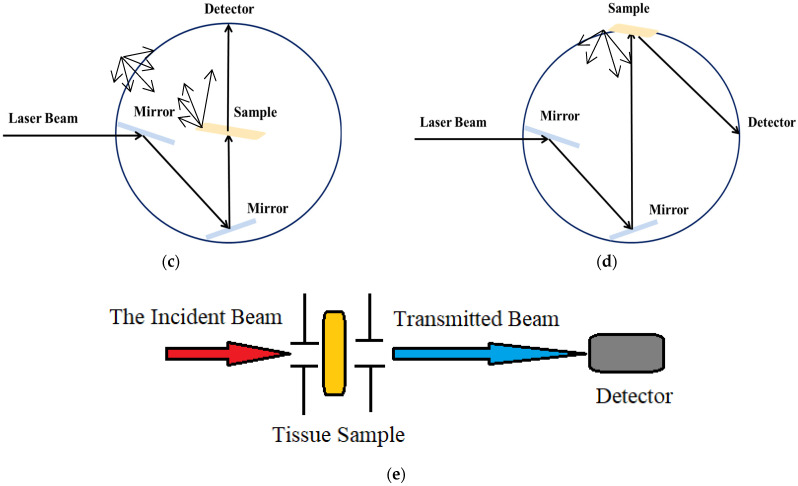
Integrating sphere-based optical configurations for the total transmittance *T*_t_ (**a**) and diffuse reflectance *R*_d_ (**b**) measurements; schematics of the total transmittance; (**c**) and diffuse reflectance (**d**) modes and (**e**) collimated transmittance.

Tissue diffuse transmittance and reflectance were measured using a single integrating sphere and CCD (TCD1304AP) connected to STDFSM digital fiber spectrometer through a data collecting optical fiber used in the experiment. Two different geometrical arrangements were implemented for measuring the total transmittance *T*_t_ and diffuse reflectance *R*_d_, as illustrated in Figure 2. Because there was no hole to allow the collimated beam to escape the integrating sphere, the collimated transmittance *T*_c_ was measured in a separate optical arrangement (outside the sphere) using a laser source, pinholes, and detector, as illustrated in Figure 2e. Accordingly, the diffuse transmittance *T*_d_ is calculated as *T*_d_ = *T*_t_ − *T*_c_. Finally, the collected data were then applied in the Kubelka–Munk model for estimation of the absorption and scattering coefficients.

### 2.4. Refractive Index Measurements

**Figure 3:**
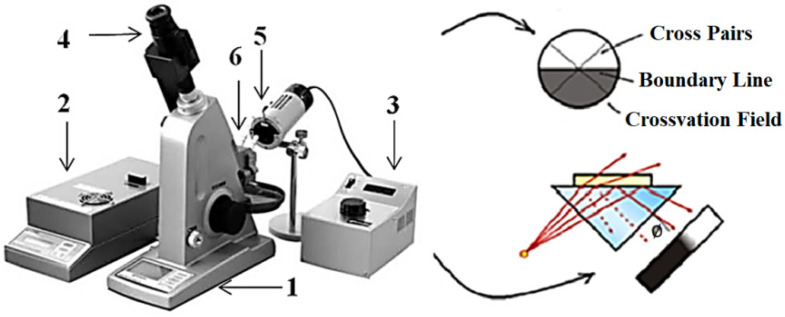
Schematics of the Abbe refractometer (Atago, Japan), which consists of the following: (1) electronic refractometer, (2) power supply, (3) multi-wavelength light source, (4) the eyepiece imager for measurements in the NIR region, (5) interference filter, and (6) sample place.

The refractive index of the tissue samples was measured using the multi-wavelength Abbe refractometer (Atago, Tokyo, Japan) (Figure 3). The RI was measured for samples of rabbit head tissues ex vivo after dividing the head into three types of tissues with an average thickness of the skin of 1.1 mm, skull of 1 mm, and brain of 3 mm. The multi-wavelength refractometer Abbe permits measuring the RI in the wavelength range of 450 to 1550 nm with an exactness of 0.0002. The functioning principle of the refractometer technique depends on deciding the critical angle of the total reflectance, where the occurrence light waves are totally reflected at a 90-degree angle to the ordinary position. The total inside reflectance method was applied for estimation of the RI of biological tissue, which is described by high light scattering and absorption. We carried out the measurements to calculate the optical parameter RI of the rabbit head tissue at wavelengths of 532, 660, 785, and 980 nm.

### 2.5. Estimation of Optical Properties

**Figure 4:**
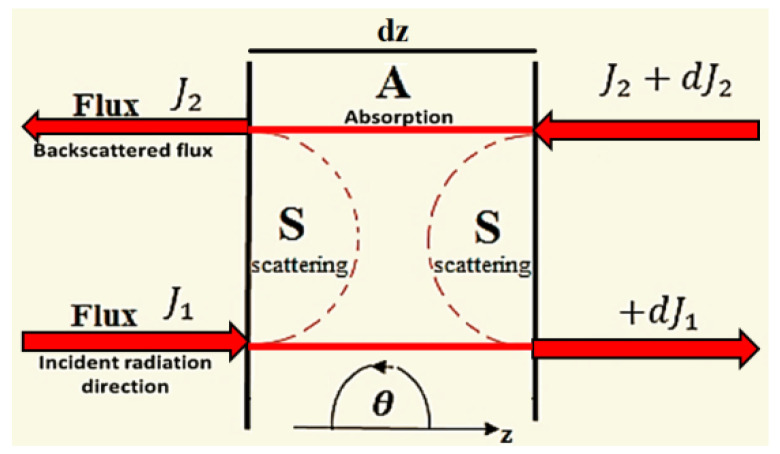
The calculation of two radiation fluxes in the Kubelka–Munk model.

Kubelka–Munk’s (KM) model permits two inside radiation fluxes through the tissue, one flux in the direction of the incident beam J_1_ and the other in the backscattered direction J_2_; the essential flux spreads in a similar direction as the incident radiation and the other flux engenders conversely, as displayed in Figure 4. Two coefficients ($A_{\mathrm{KM}\;}$ and $S_{\mathrm{KM}}$) are proposed to show the absorption and scattering of the diffuse radiation, individually [23,24,25,26,27,28,29]:

(1) $$\frac{dJ_{1}}{dz}=-S_{\mathrm{KM}}\;J_{1}-A_{\mathrm{KM}}\;J_{1}+\;S_{\mathrm{KM}}\;J_{2},$$

(2) $$\frac{dJ_{2}}{dz}=-S_{\mathrm{KM}}\;J_{2}-A_{\mathrm{KM}}\;J_{2}+\;S_{\mathrm{KM}}\;J_{1},$$

where z alludes to the primary direction of the incident radiation, as indicated by Kottler [23]. The Kubelka–Munk coefficients are connected so as to measure the diffuse transmittance $T_{d}$ and diffuse reflectance $R_{d}$ as [23,24]:

(3) $$R_{d}=\frac{\sinh(S_{\mathrm{KM}}yd)}{x\cosh\left(S_{\mathrm{KM}}yd\right)+y\sinh(S_{\mathrm{KM}}yd)\;},$$

(4) $$T_{d}=\frac{y}{x\cosh\left(S_{\mathrm{KM}}yd\right)+y\sinh(S_{\mathrm{KM}}yd)\;},$$

where d is the optical thickness of the piece to be considered, and parameters x and y are expressed as follows:

(5) $$x=\frac{1+R_{d}^{2}-T_{d}^{2}}{2R_{d}},$$

(6) $$y=\sqrt{x^{2}-1\;}.$$

The Kubelka–Munk model is a unique instance of the supposed multi-flux theory, where the transport equation is changed into a matrix differential equation by considering the radiance at numerous discrete angles [25]. The convenience of applying the Kubelka–Munk method arises from the fact that the absorption coefficient $\mu_{a}$ and reduced scattering coefficient $\mu_{s}^{\prime }$ can be directly calculated from the measured diffuse transmittance $T_{d}$ and diffuse reflectance $R_{d}$, i.e., as follows:

(7) $$S_{\mathrm{KM}}=\frac{1}{yd}\;\ln\left[\frac{1-R_{d}\left(x-y\right)}{T_{d}}\right],$$

(8) $$A_{\mathrm{KM}}=\left(x-1\right)S_{\mathrm{KM}}.$$

Then, the relation between $S_{\mathrm{KM}}$ and $A_{\mathrm{KM}}$ and the scattering $\mu_{s}\;$ and absorption $\mu_{a}$ coefficients of the sample can be expressed as follows:

(9) $$A_{\mathrm{KM}}=2\mu_{a},\;S_{\mathrm{KM}}=\frac{3}{4}\mu_{s}\left(1-g\right)-\frac{1}{4}\mu_{a},$$

where *g* represents the scattering anisotropy factor and $\mu_{s}^{\prime }=\mu_{s}\left(1-g\right)$.

In addition, the optical penetration depth δ can be estimated using the following equation, which is valid in the diffusion approximation: [28]

(10) $$\delta =\frac{1}{\sqrt{3\mu_{a}\left(\mu_{a}+\mu_{s}^{\prime }\right)}}\;.$$

Regarding the determination of the refractive index (RI) of the studied tissues, the dispersion is represented as follows:

(11) $$n^{*}\left(\lambda \right)=n\left(\lambda \right)+ik\left(\lambda \right),$$

where $n\left(\lambda \right)$ is the real part of the complex RI $n^{*}\left(\lambda \right)$ and $k\left(\lambda \right)$ is its imaginary part which is related to the tissue absorption coefficient by the Kramers–Kronig relations, as follows [29]:

(12) $$k\left(\lambda \right)=\frac{\lambda \mu_{a}\left(\lambda \right)}{4\pi }\;.$$

Additionally, the Fresnel equation connects the specular reflectance at a normal incidence $R\left(\lambda \right)$ and the tissue RI as follows [30,31,32,33,34,35,36,37,38,39]:

(13) $$R_{F}\left(\lambda \right)=\frac{{\left(n\left(\lambda \right)-1\right)}^{2}+\;k{\left(\lambda \right)}^{2}}{{\left(n\left(\lambda \right)+1\right)}^{2}+\;k{\left(\lambda \right)}^{2}}.$$

In general, the RI of the tissues samples is measured at discrete wavelengths using multi-wavelength Abbe refractometers [40] or by utilizing the total internal reflectance method with different lasers [41]. When the RI values are estimated, the tissue dispersion for that wavelength range is calculated by apply the experimental RI data with equations such as the Cauchy (Equation (14)), the Conrady (Equation (15)), or the Cornu (Equation (16)) equations [41,42,43]:

(14) $$n_{\mathrm{tissue}}\left(\lambda \right)=A+\frac{B}{\lambda^{2}}+\frac{C}{\lambda^{4}}\;,$$

(15) $$n_{\mathrm{tissue}}\left(\lambda \right)=A+\frac{B}{\lambda }+\frac{C}{\lambda^{3.5}}\;,$$

(16) $$n_{\mathrm{tissue}}\left(\lambda \right)=A+\frac{B}{\left(\lambda -C\right)},$$

where, A, B, and C are the Cauchy, the Conrady, and the Cornu parameters, respectively. Using the Kramers–Kronig relations, which were developed for non-scattering materials, a broader dispersion can be calculated from μ_a_(λ) if the calculated tissue dispersion is not available for the entire wavelength range of interest. Equation (12) is the first one to be used to obtain the imaginary part of tissue dispersion (κ(λ)) [41,44]. When known, κ(λ), the dispersion that corresponds to the real part of the refractive index (RI), can be calculated from [41,44]:

(17) $$n_{\mathrm{tissue}}\left(\lambda \right)=1+\frac{2}{\pi }\int_{0}^{\infty }\frac{\lambda_{1}}{\Lambda }\;\frac{\lambda_{1}}{\Lambda^{2}-\lambda_{1}^{2}}K\left(\Lambda \right)d\Lambda$$

where Λ is the integrating variable over the wavelength space and λ_1_ is a decent wavelength that can be adapted for better matching of the determined scattering to the one obtained from the discrete experimental information. When the expansive band tissue scattering is determined through Equations (12) and (17), we can choose discrete values from it to use in the inverse adding-doubling (IAD) simulations.

Running (IAD) simulations will generate $\mu_{s}^{\prime }$ values are then given with the equation [28,42]:

(18) $$\mu_{s}^{\prime }\left(\lambda \right)=a\left[f_{\mathrm{Ray}}{\left(\frac{\lambda }{500\;\mathrm{nm}}\right)}^{-4}+\left(1-f_{\mathrm{Ray}}\right){\left(\frac{\lambda }{500\;\mathrm{nm}}\right)}^{-b_{Mie}}\right],$$

where *a* is the scaling factor that addresses the value of $\mu_{s}^{\prime }$ at 500 nm, *f*_Ray_ is the Rayleigh scattering fraction, and *b_Mie_* represents the mean size of the Mie scatterers. These parameters can be achieved when the discrete $\mu_{s}^{\prime }$ values that were produced through IAD are given, using Equation (18).

Such a spectrum can be joined with the μ_s_ spectrum that is determined using the measurements of collimated transmittance $T_{c}\left(\lambda \right)$ = *T*_t_ − *T*_d_ and thickness *d* [28,42]:

(19) $$\mu_{s}\left(\lambda \right)=\frac{\ln\left[T_{c}\left(\lambda \right)\right]}{d}-\mu_{a}\left(\lambda \right).$$

To obtain the wavelength reliance of the tissue dispersing anisotropy factor (*g*(λ)), both scattering coefficients are used: [45]

(20) $$g\left(\lambda \right)=1-\frac{{\;\mu }_{s}^{\prime }\left(\lambda \right)}{\mu_{s}\left(\lambda \right)}$$

In general, *g*(λ) gives a rising exponential behaviour expanding wavelength in the UV–NIR range [1], which can be described mathematically using Equation (21) [45] or Equation (22) [46].

(21) $$g\left(\lambda \right)=a+b\left[1-\exp\left(\frac{\lambda -c}{d}\right)\right],$$

(22) $$g\left(\lambda \right)=a\;\exp\left(b\lambda \right)+c\;\exp\left(d\lambda \right),$$

where a, b, c, and d are the parameters that are acquired during the gives of *g* data. This estimation system depends just on IAD simulations to obtain discrete ($\mu_{s}^{\prime }$) values [47].

### 2.6. Monte-Carlo Simulation for Modeling of Light Diffusion

**Figure 5:**
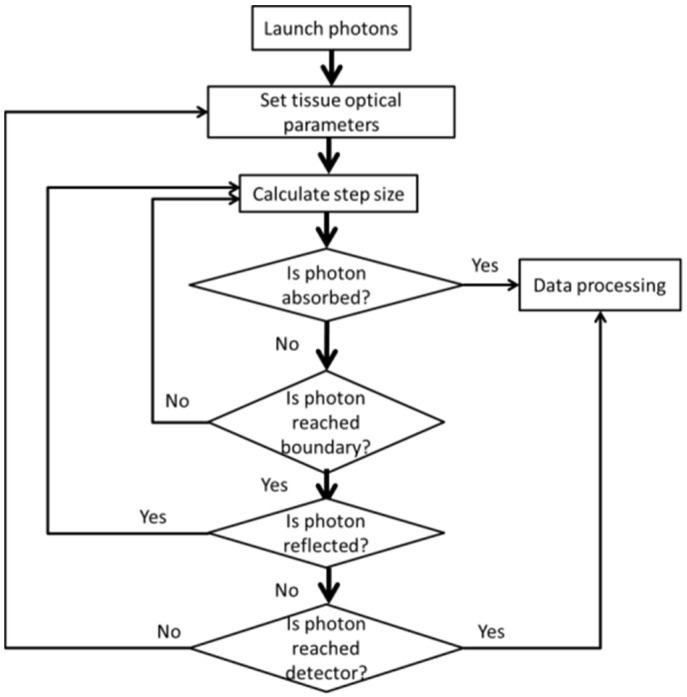
Flow chart of the Monte-Carlo method.

Monte-Carlo (MC) is a very common computational modeling algorithm used to simulate light spread in one- or multi-layer biological slabs [31,32,33]. As an advanced method, the MC execution needs input data regarding the analyzed piece. Consequently, each layer is defined by its RI, scattering anisotropy factor, scattering coefficient, absorption coefficient, and thickness. The simulation begins by submitting the fitting number of photons and the tracing process starts as per the predefined step size and limit conditions. The MC simulation process is summarized in the following flowchart (Figure 5).

To use the simulation, five parameters with respect to the sample must be known. The simulation output gives the worth of the diffuse reflectance that ought to be obtained from the experimental estimations. In our experimental, the achieved thickness and optical parameters of the samples were introduced to MC Multi-Layered (MCML) [33], expecting matched boundary conditions in order to achieve the values of the diffuse reflectance in each case, hence validating our results. The simulation was applied using the Matlab R2018a program.

Where all the measurements of the parameters that we obtained from our experiment were entered in the Matlab (2018) program, and after applying the Monte Carlo program code, the photons were distributed—approximately 100,000 photons in the tissues—which were then measured after interacting with them, according to the data and the angles of deviation in the photon path when scattering occurred. This statistic depends on calculating the spread of a large number of photons. As a result, this method requires a long computational time.

### 2.7. Simulating Fluence Distribution

The distribution of the optical fluence of the head tissue layers was determined by utilizing the finite element solution of the diffusion equation. The fluence rate is determined from the following equation [26]:

(23) $$\frac{\partial \Phi \left(\vec{r},t\right)}{c\partial t}+\mu_{a}\Phi \left(\vec{r},t\right)-\nabla \left[D\nabla \Phi \left(\vec{r},t\right)\right]=S\left(\vec{r},t\right),$$

where $D=\frac{1}{3\left(\mu_{a}+\mu_{s}^{\prime }\right)}$ is the tissue diffusion coefficient, $S\left(\vec{r},t\right)$ represents the source term, and $\Phi \left(\vec{r},t\right)$ is the fluence rate.

To solve diffusion equations numerically, which is a forward problem, the finite element method (FEM) can be used [26]. In this case, the fluence distribution $\Phi \left(\vec{r},t\right)$ is gained in the domain as a function F of optical properties μ(*r*), as follows:

(24) $$\Phi =F\left(\mu \right),$$

where $\mu =\left[\mu_{a},\;D\right]$.

In diffuse optical imaging, the image reconstruction process requires the solution of the inverse problem in which the distribution of $\Phi \left(\vec{r},t\right)$ at the boundary is given, while optical properties of the domain are unknown [27]; this can be represented by the following:

(25) $$\mu =F^{-1}\left(\Phi \right)$$

Solving the inverse problem requires minimization of the error function χ^2^, which can be calculated as follows:

(26) $$\chi^{2}=\frac{1}{2}\overset{M}{\underset{J=1}{\sum }}{\left(\Phi_{\mathrm{meas}}-F\left(\mu \right)\right)}^{2}\;,$$

where $\Phi_{\mathrm{meas}}$ is the fluence measurements at the boundaries and $F\left(\mu \right)$ is the calculated fluence ($\Phi_{\mathrm{calc}}$) using the forward model. In the present work, the finite element method (FEM) using the diffusion equation was implemented to get up fluence rate distribution images at the boundary of the sample surfaces using Matlab R2018b and COMSOL Multiphysics 5.4 program software.

In COMSOL Multiphysics, the diffusion of Equation (23) in the steady-state can be presented using the Helmholtz equation:

(27) $$\nabla \left(-c\nabla u\right)+au=f$$

Identifying the parameters from Equation (23) yields the following:

(28) $$\begin{array}{c} u=\Phi ,\;a=\mu_{a},\\ c=D,\;f=S. \end{array}$$

**Figure 6:**
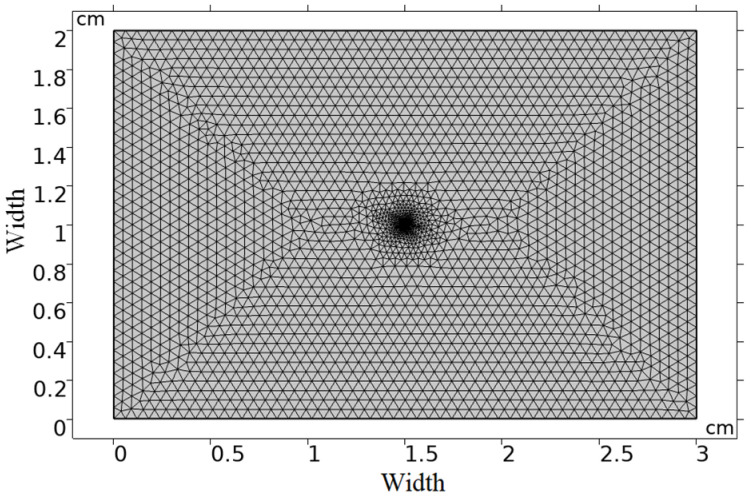
Finite element mesh and geometry for the fluence rate determination.

The executed model was a rectangle of 3 cm × 2 cm in width. A point source addressing the laser source was placed at a location of (1.5, 1) to simulate the laser source position in the integrating sphere configuration, as illustrated in Figure 6.

## 3. Results and Discussion

### 3.1. Determination of Scattering and Absorption Coefficients

**Figure 7:**
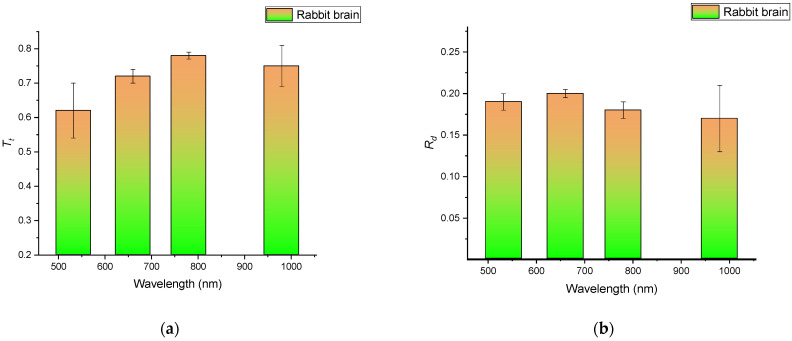

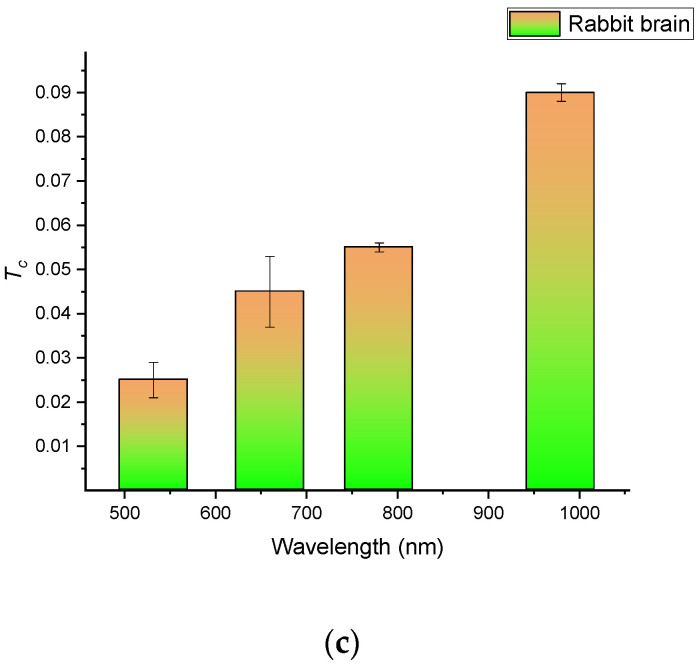
The measured total transmittance *T*_t_ (**a**), diffuse reflectance *R*_d_ (**b**), and collimated transmittance *T*_c_ (**c**) of the rabbit brain samples were prepared with an average thickness of 0.5 mm. *T*_t_ + *R*_t_ + *A* = 1, where *A* is the relative absorbed intensity *A* = 1 − exp(−μ_a_*d*). As μ_a_*d* was sufficiently small in our study, *A* ≅ 1 − [1 − μ_a_*d*] = *μ_a_d*, *T*_t_ = 1 − (*R*_d_ + *R*_F_) − μ_a_*d*.

The tissue sample total transmittance *T*_t_, collimated transmittance *T*_c_, and diffuse reflectance *R*_d_ were measured using an integrating sphere and two-pinhole arrangement, as illustrated in Figure 2. For the RI measurements, several samples of rabbit brain tissue were prepared with a 3 mm thickness. For the spectral measurements, 10 samples were prepared with a 0.5 mm thickness. The results of the measurements for the freshly native rabbit brain at wavelengths of 532, 660, 785, and 980 nm are presented in Figure 7. Accordingly, reduced scattering and absorption coefficients were extracted for the freshly native rabbit brain, skin, and skull at wavelengths of 532, 660, 785, and 980 nm. The obtained values were compared with previously published data, as summarized in Table 1.

**Figure 8:**
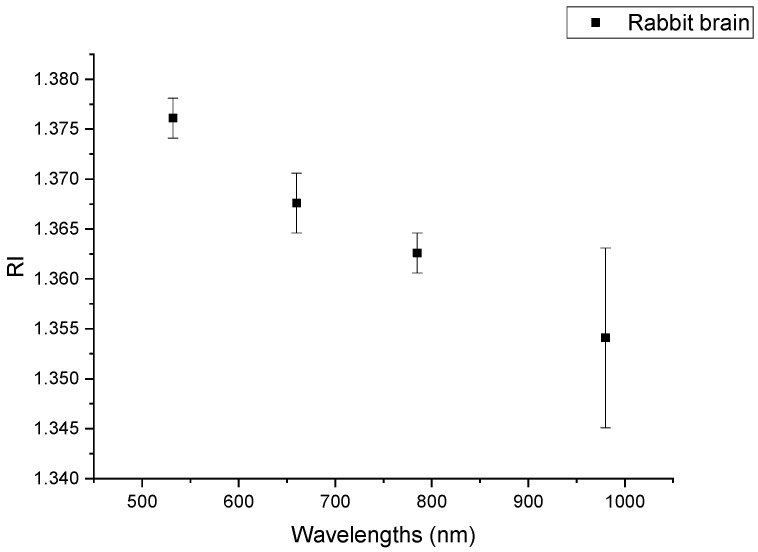
The (RI) refractive index of the rabbit brain tissue with the standard deviation.

The data for the (RI) refractive index of all rabbit head tissue types measured independently are presented in Table 1, and Figure 8 shows the (RI) refractive index of the rabbit brain tissue with the standard deviation.

**Figure 9:**
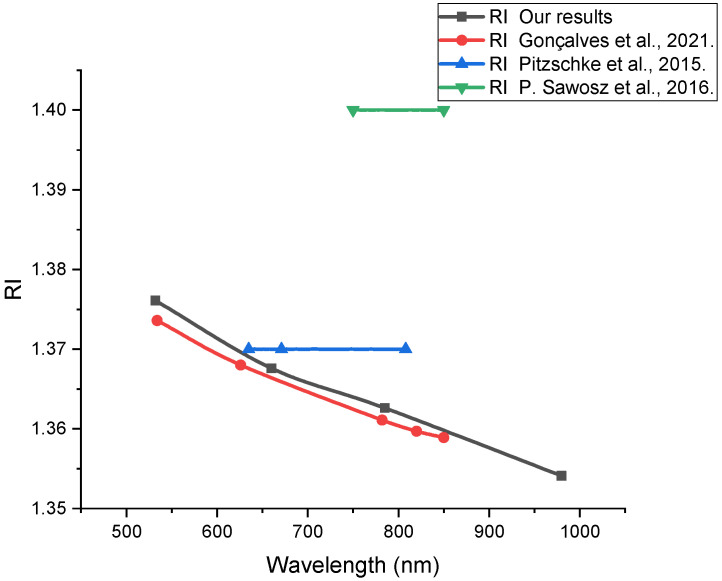
Wavelength dispersion of the refractive index of the rabbit brain, measured in this work (squares), data from Gonçalves et al. [29] (circles), data from Pitzschke et al. [37] (upward triangles), and data from P. Sawosz et al. [39] (downward triangles).

The data for the (RI) refractive index compared with the data from the literature are presented in Figure 9.

**Figure 10:**
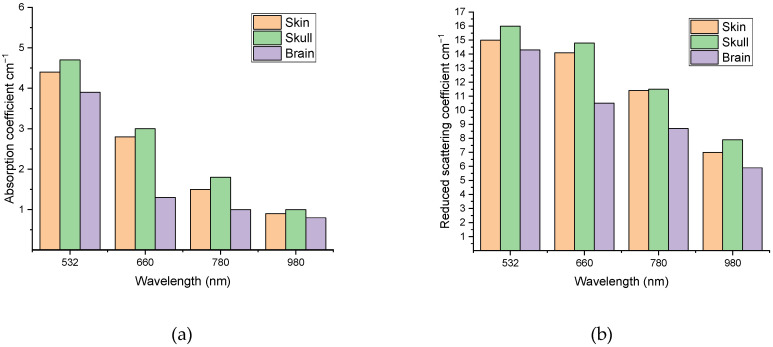

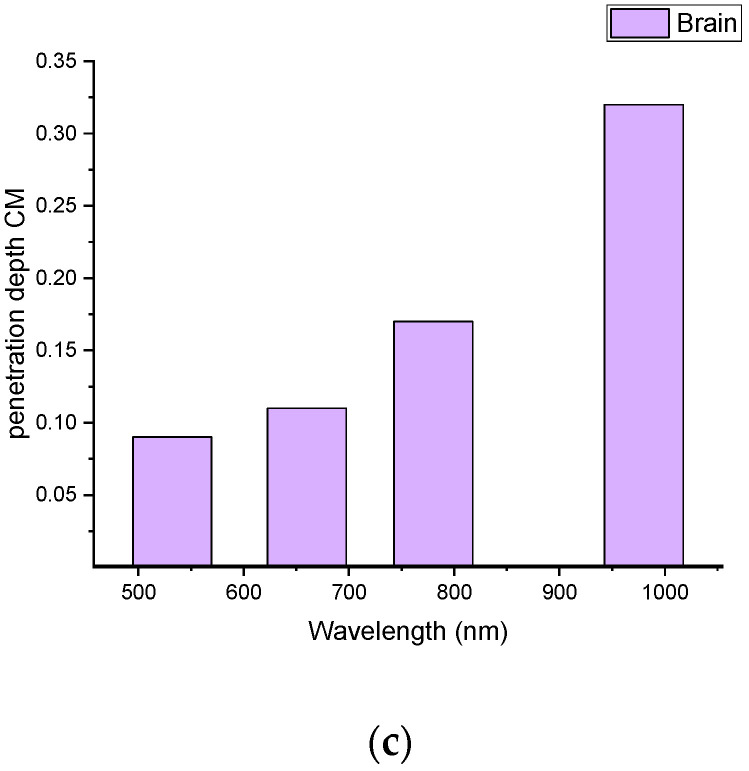
Variation in the calculated optical parameters at different wavelengths for all three investigated types of rabbit head tissues: absorption coefficient (**a**), reduced scattering coefficient (**b**), and penetration depth of the rabbit brain using Equation (10) (**c**).

Figure 10 illustrates the variation in the calculated optical properties and penetration depth at the studied wavelengths.

**Figure 11:**
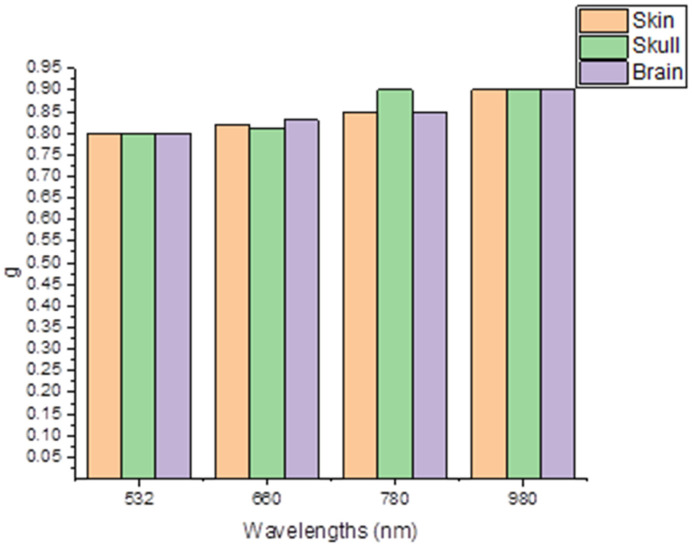
Anisotropy factor (*g*) measured for all of the tissue types.

In our implementation, we measured the anisotropy factor (*g*) of all of the tissue types. These data are presented in Figure 11.

**Figure 12:**
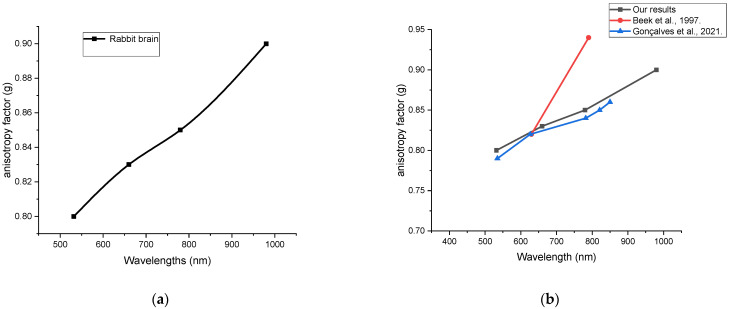
Anisotropy factor was measured (**a**) in rabbit brain tissue and (**b**) compared our data with data from the literature, there was a difference between this and the variance factor measured for Beek et al. [36] In contrast with Gonçalves et al. [29], which was closer to our measurements.

The proportion of the contrast factor in Figure 11 differs slightly in the scalp compared with in the skull, as well as the brain for the rabbit head, as well as with the different wavelengths used in our experiment. Therefore, little variation was noted between them, while in Figure 12 which compared our data with data from the literature, there was a difference between this and the variance factor measured for [36] in contrast with [29], which was closer to our measurements.

**Figure 13:**
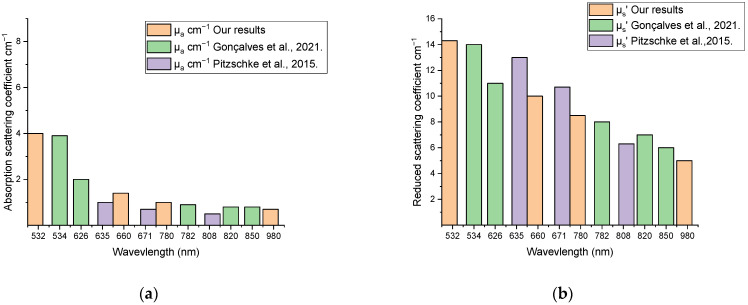
Comparison with the data from the literature Gonçalves et al. [29] and Pitzschke et al. [37] measured using the absorption coefficient μ_a_ (**a**) and reduced scattering coefficient $\mu_{s}^{\prime }$ (**b**) of the rabbit brain.

In the present study, ex vivo optical parameters of the rabbit skin, skull, and brain were determined and compared with data from the literature (Figure 13). The parameters were obtained for four laser wavelengths ranging from visible to NIR optical regions. The proposed results showed similarly reduced scattering coefficients of the skull at 532 nm to that published by Soleimanzad et al. [34] for mice skull, and Firbank et al. [35] for pig skull at 660 and 780 nm, while there were some differences in the absorption coefficient values. Regarding the skin samples, the optical parameters obtained at 660 were a little higher than at 780 nm, which followed almost the same behavior compared with those obtained by Beek et al. [36] at 630 and 790 nm. Moreover, the obtained optical parameters of the rabbit brain were comparable to those obtained by Pitzschke et al. [37] at near wavelengths with some variations within the acceptable range. In principle, the sample preparation method, sample thickness, experimental technique, and the employed mathematical model could be reasons for the relatively spread values for μ_a_ and $\mu_{s}^{\prime }$ in the literature [38].

### 3.2. Modeling the Fluence Rate Distribution

**Figure 14:**
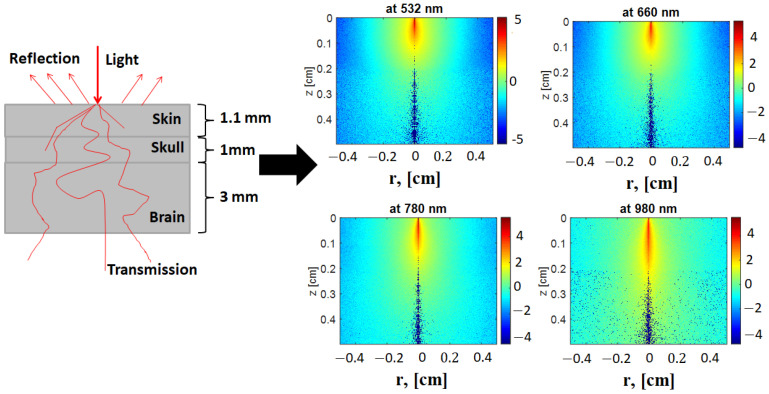
The distribution of the optical fluence rate (logarithm of the fluence values) in the model containing the three layers (skin, skull, and brain).

Using the MCML source code, the fluence rate distribution inside each separate tissue sample was simulated at each studied wavelength, as demonstrated in Figure 14. The fluence rate distributions through a block containing the three tissue layers (skin, skull, and brain) were obtained for all four wavelengths.

As shown in Figure 14, the fluence rate distribution inside the tissue increased at longer wavelengths for each tissue type. The maximum fluence rate was obtained at 980 nm because of the low scattering and better transmittance. The simulation results showed a clear increase in the fluence distribution inside the three-layered model at 980 nm, followed by 785 nm, 660 nm, and 532 nm.

**Figure 15:**
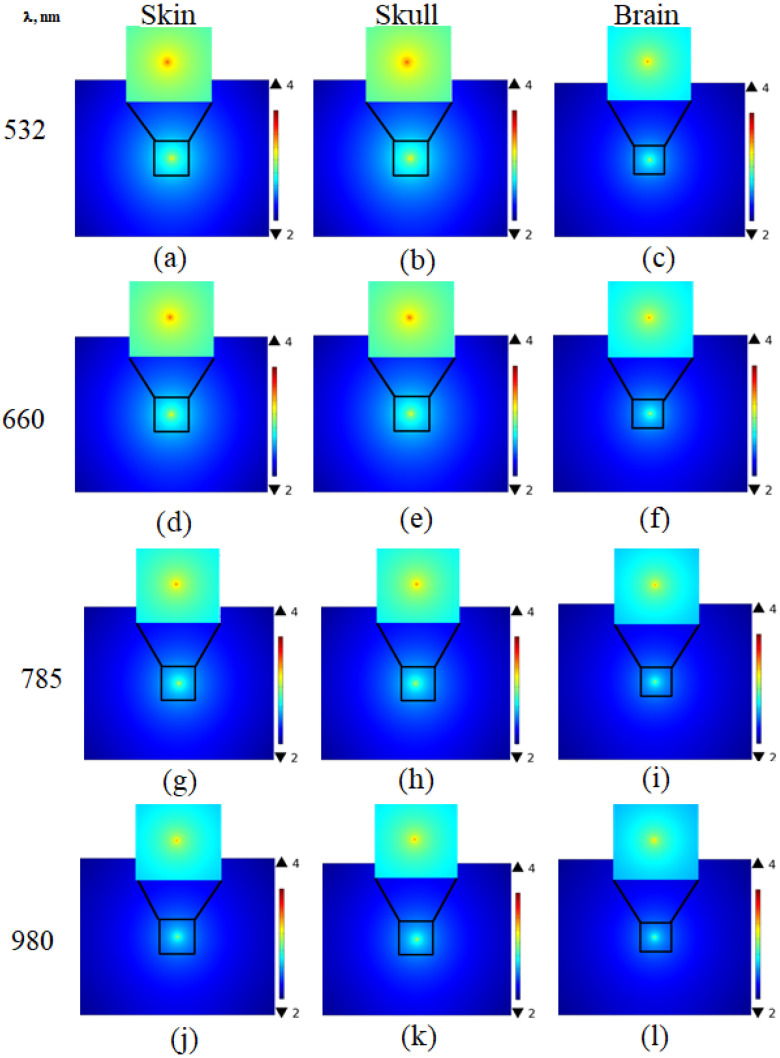
The fluence rate distributions at the surface outer of the skin (**a**), skull (**b**), and brain (**c**) at 532 nm; skin (**d**), skull (**e**), and brain (**f**) at 660 nm; skin (**g**), skull (**h**) and brain (**i**) at 785 nm; and skin (**j**), skull (**k**) and brain (**l**) at 980 nm.

The fluence rate at the outer surface of the sample was based on the finite element method (FEM) solution of the diffusion equation via COMSOL multiphysics software. The obtained results are presented in Figure 15. The fluence rate at the tissue surface became less diffused for longer wavelengths as a result of the low scattering and better penetration.

The obtained optical parameter values of the rabbit head tissue samples were introduced, through Equation (23), to the diffusion of light propagation in biological tissue to investigate the change in the fluence rate distribution at the tissue surface as a result of the change in optical parameters. Figure 14 and Figure 15 show the difference in the optical fluence rate images in a model containing three layers (skin, skull, and brain) of rabbit head tissue samples at 532, 660, 785, and 980 nm wavelengths.

The difference in fluence rate values at each wavelength resulting from the change in optical parameter values introduced to the diffusion equation were used to obtain the fluence rate distribution at the sample’s surface. Varying the sample condition affected the water and blood contents, and hence affected the values of the optical parameters. Moreover, it can be observed from Figure 15 that the fluence rate distribution at the surface of the samples changed with the tissue type due to the change in their optical properties.

## 4. Conclusions

Recent technological advances in photonics have prompted extensive advancements in the improvement of imaginative strategies and systems for clinical and functional optical imaging, phototherapy, and laser surgery. Intensive studies and the development of biomedical optical methods and techniques have stimulated great interest in the quantitative assessment of the optical properties of tissues. The optical properties of the head tissue depend on the laser wavelength, and the delivery of radiation to the target in the depth of the tissue depends on the fluence rate on the surface and the thickness of the head tissue layers. The Kubelka–Munk model and Monte-Carlo modeling were used in this work to evaluate the optical properties of the skin, skull, and brain tissues of the rabbit head from measurements of diffuse reflectance and the transmittance of tissue samples using a single integrating sphere. The obtained data for the optical properties were close to the previously published data and significantly supplement them.

As the attenuation of radiation at the considered wavelengths is quite strong, it is necessary to use methods of optical clearing of the upper layers of the head tissue. The experimental and theoretical methods developed in this study should be used for analyzing the distribution of the laser fluence rate in the case of optical clearing, which is used to reduce the radiation density in the upper layers of the head in order to avoid strong heating when exposed to intense laser beams.

In conclusion, a method based on measuring the optical properties of biological tissues using integrative sphere and optical fluorescence distributions on tissue surfaces has been applied as a new diagnostic tool. In this method, a combination of the Kubelka–Munk model and Monte-Carlo model was used to calculate the optical parameters of different samples of biological tissues from the measured values of diffuse reflectance and transmittance using the diffusion equation. The measurements were carried out using an experimental setup based on the refractometer and integration sphere in rabbit head tissues at selected laser wavelengths. The currently proposed method is suitable for in vitro measurements; however, it can be upgraded via the use of optical fiber probes to be suitable for in vivo study. The results obtained here are promising because the fluency rate distribution images have discriminatory features between different tissue types. Therefore, this method can be used in the diagnosis and differentiation of biological tissues.

## Figures and Tables

**Table 1:** The obtained optical parameters were compared with the relevant data from the literature.

Literature	Tissue	λ, nm	Optical Parameters			
			μ_a_, cm^−1^	$\mu_{s}^{\prime }$, cm^−1^	*g*	*n*
Soleimanzad et al. [34]	Mice skull (ex vivo)	532	14 ± 2	25 ± 1.5	-	-
		660	5 ± 0.5	25 ± 2.5	-	-
		705	4.7 ± 0.7	22.9 ± 1.2	-	-
Firbank et al. [35]	Pig skull (ex vivo)	660	0.3 ± 0.025	23.7 ± 1.2	~0.93	-
		780	0.25 ± 0.025	18.5 ± 1.2	~0.93	-
Beek et al. [36]	Rabbit skin (in vitro)	630	0.94 ± 0.13	40 ± 2.2	0.812 ± 0.017	-
		790	0.7 ± 0.07	18.4 ± 0.5	0.940 ± 0.003	-
Pitzschke et al. [37]	Rabbit brain (in vivo)	635	1.1 ± 0.2	12.8 ± 0.1	0.92	1.37
		671	0.8 ± 0.2	10.7 ± 0.9	0.92	1.37
		808	0.5 ± 0.1	6.2 ± 0.5	0.92	1.37
Gonçalves et al. [29]	Rabbit brain (cerebral cortex, ex vivo)	401.4	~9.5	~20	~0.72	1.3850 ± 0.0053
		534.6	~3.9	~14	~0.79	1.3736 ± 0.0058
		626.6	~2.1	~11	~0.82	1.3680 ± 0.0045
		782.1	~0.9	~8	~0.84	1.3611 ± 0.0050
		820.8	~0.8	~7	~0.85	1.3597 ± 0.0066
		850	~0.8	~6	~0.85	1.3589 ± 0.0076
P. Sawosz et al. [39]	Human scalp	750	0.125	13.0	-	1.4
		850	0.180	13.0	-	1.4
	Human skull	750	0.082	12.0	-	1.4
		850	0.118	12.0	-	1.4
	Human CSF	750	0.025	10.0	-	1.4
		850	0.041	10.0	-	1.4
	Human brain	750	0.156	12.5	-	1.4
		850	0.200	12.5	-	1.4
This study	Rabbit skin (ex vivo)	532	4.4 ± 0.6	15.0 ± 0.2	0.80	1.3769 ± 0.002
		660	2.8 ± 0.4	14.1 ± 0.2	0.82	1.3691 ± 0.002
		785	1.5 ± 0.8	11.4 ± 0.3	0.85	1.3631 ± 0.003
		980	0.8 ± 0.6	6.0 ± 0.2	0.90	1.3551 ± 0.002
	Rabbit skull (ex vivo)	532	4.7 ± 1.5	16.0 ± 0.5	0.80	1.3784 ± 0.005
		660	3.0 ± 0.4	14.8 ± 0.1	0.81	1.3695 ± 0.001
		785	1.7 ± 0.1	11.5 ± 0.4	0.90	1.3637 ± 0.004
		980	0.9 ± 0.4	7.2 ± 0.2	0.90	1.3596 ± 0.002
	Rabbit brain (ex vivo)	532	4.0 ± 0.3	14.3 ± 0.9	0.80	1.3761 ± 0.002
		660	1.2 ± 0.1	10.5 ± 0.3	0.83	1.3676 ± 0.003
		785	1.0 ± 0.7	8.7 ± 0.2	0.85	1.3626 ± 0.002
		980	0.8 ± 0.6	5.9 ± 0.2	0.90	1.3541 ± 0.009

## Data Availability

Data supporting reported results can be provided by the authors for request.

## References

[1] Bashkatov A.N., Berezin K.V., Dvoretskiy K.N., Chernavina M.L., Genina E.A., Genin V.D., Tuchin V.V. (2018). Measurement of tissue optical properties in the context of tissue optical clearing. J. Biomed. Opt..

[2] Watson J.R., Martirosyan N., Lemole G.M., Trouard T.P., Romanowski M. (2018). Intraoperative brain tumor resection with indocyanine green using augmented microscopy. J. Biomed. Opt..

[3] Macdonald C.M., Arridge S., Powell S. (2020). Efficient inversion strategies for estimating optical properties with Monte Carlo radiative transport models. Biomed. Opt..

[4] Byrd B.K., Marois M., Tichauer K.M., Wirth D.J., Hong J., Leonor J.P., Davis S.C. (2019). First experience imaging short-wave infrared fluorescence in a large animal: Indocyanine green angiography of a pig brain. J. Biomed. Opt..

[5] Salehpour F., Cassano P., Rouhi N., Hamblin M.R., De Taboada L., Farajdokht F., Mahmoudi J. (2019). Penetration Profiles of Visible and Near-Infrared Lasers and Light-Emitting Diode Light through the Head Tissues in Animal and Human Species: A Review of Literature. Photobiomodulation Photomed. Laser Surg..

[6] Gioux S., Mazhar A., Cuccia D.J. (2019). Spatial frequency domain imaging in 2019: Principles, applications, and perspectives. J. Biomed. Opt..

[7] Ayaz H., Izzetoglu M., Izzetoglu K., Onaral B., Dor B.B. (2021). Early diagnosis of traumatic intracranial hematomas. J. Biomed. Opt..

[8] Wang J., Lin J., Chen Y., Welle C.G., Pfefer T.J. (2019). Phantom-based evaluation of near-infrared intracranial hematoma detector performance. J. Biomed. Opt..

[9] Zhao H., Wang Y., Chen L., Shi J., Ma K., Tang L., Chen T. (2018). High-sensitivity terahertz imaging of traumatic brain injury in a rat model. J. Biomed. Opt..

[10] Meitav O., Shaul O., Abookasis D. (2018). Spectral refractive index assessment of turbid samples by combining spatial frequency near-infrared spectroscopy with Kramers–Kronig analysis. J. Biomed. Opt..

[11] Pasarikovski C.R., Cardinell J., Yang V.X. (2019). Perspective review on applications of optics in cerebral endovascular neurosurgery. J. Biomed. Opt..

[12] Madsen S.J., Christie C., Huynh K., Peng Q., Uzal F.A., Krasieva T.B., Hirschberg H. (2018). Limiting glioma development by photodynamic therapy-generated macrophage vaccine and allo-stimulation: An in vivo histological study in rats. J. Biomed. Opt..

[13] Habimana-Griffin L., Ye D., Carpenter J., Prior J., Sudlow G., Marsala L., Achilefu S. (2020). Intracranial glioma xenograft model rapidly reestablish blood-brain barrier integrity for longitudinal imaging of tumor progression using fluorescence molecular tomography and contrast agents. J. Biomed. Opt..

[14] Zemp R. (2018). Estimation of the cerebral metabolic rate of oxygen consumption using combined multi-wavelength photoacoustic microscopy and Doppler micro-ultrasound. J. Biomed. Opt..

[15] Ma L., Fei B. (2021). Comprehensive review of surgical microscopes: Technology development and medical applications. J. Biomed. Opt..

[16] Tedford C.E., DeLapp S., Jacques S., Anders J. (2015). Quantitative analysis of transcranial and intraparenchymal light penetration in human cadaver brain tissue. Lasers Surg. Med..

[17] Strangman G.E., Zhang Q., Li Z. (2014). Scalp and skull influence on near-infrared photon propagation in the Colin27 brain template. NeuroImage.

[18] Souza-Barros L., Dhaidan G., Maunula M., Solomon V., Gabison S., Lilge L., Nussbaum E.L. (2018). Skin color and tissue thickness effects on transmittance, reflectance, and skin temperature when using 635 and 808 nm lasers in low-intensity therapeutics. Lasers Surg. Med..

[19] Kim S., Jeong S. (2014). Effects of temperature-dependent optical properties on the fluence rate and temperature of biological tissue during low-level laser therapy. Lasers Med. Sci..

[20] Angell-Petersen E., Hirschberg H., Madsen S.J. (2007). Determination of fluence rate and temperature distributions in the rat brain; implications for photodynamic therapy. J. Biomed. Opt..

[21] Hamdy O., El-Azab J., Al-Saeed T.A., Hassan M.F., Solouma N.H. (2017). A Method for Medical Diagnosis Based on Optical Fluence Rate Distribution at Tissue Surface. Materials.

[22] Hamdy O., Fathy M., Al-Saeed T.A., El-Azab J., Solouma N.H. (2017). Estimation of optical parameters and fluence rate distribution in biological tissues via a single integrating sphere optical setup. Opt.-Int. J. Light Electron Opt..

[23] Kottler F. (1960). Turbid Media with Plane-Parallel Surfaces. J. Opt. Soc. Am..

[24] Markolf N.H. (2007). Laser-Tissue Interactions: Fundamentals and Applications.

[25] Ishimaru A. (1996). Wave Propagation and Scattering in Random Media.

[26] Boas D.A., Pitris C., Ramanujam N. (2011). Handbook of Biomedical Optics.

[27] Arridge S.R., Schweiger M. (1995). Photon-measurement density functions. Part 2: Finite-element-method calculations. Appl. Opt..

[28] Tuchin V.V. (2015). Tissue Optics: Light Scattering Methods and Instruments for Medical Diagnostics.

[29] Gonçalves T.M., Martins I.S., Silva H.F., Tuchin V.V., Oliveira L.M. (2021). Spectral Optical Properties of Rabbit Brain Cortex between 200 and 1000 nm. Photochem.

[30] Gienger J., Groß H., Neukammer J., Bär M. (2016). Determining the refractive index of human hemoglobin solutions by Kramers–Kronig relations with an improved absorption model. Appl. Opt..

[31] Zhu C., Liu Q. (2013). Review of Monte Carlo modeling of light transport in tissues. J. Biomed. Opt..

[32] Dolganova I.N., Neganova A.S., Kudrin K.G., Zaytsev K.I., Reshetov I.V. (2016). Monte Carlo simulation of optical coherence tomography signal of the skin nevus. J. Phys. Conf. Ser..

[33] Wang L., Jacques S.L., Zheng L. (1995). MCML-Monte-Carlo modeling of light transport in multi-layered tissues. Comput. Methods Programs Biomed..

[34] Soleimanzad H., Pain F. (2017). Optical properties of mice skull bone in the 455- to 705-nm range skull bone in the 455- to 705-nm range. J. Biomed. Opt..

[35] Firbank M., Hiraoka M., Essenpreis M., Delpy D.T. (1993). Measurement of the optical properties of the skull in the wavelength range 650–950 nm. Phys. Med. Biol..

[36] Beek J.F., Blokland P., Posthumus P., Aalders M., Pickering J.W., Sterenborg H.J.C.M., Van Gemert M.J.C. (1997). In vitro double-integrating-sphere optical properties of tissues between 630 and 1064 nm. Phys. Med. Biol..

[37] Pitzschke A., Lovisa B., Seydoux O., Haenggi M., Oertel M.F., Zellweger M., Wagnières G.A. (2015). Optical properties of rabbit brain in the red and near-infrared: Changes observed under in vivo, postmortem, frozen, and formalin-fixated conditions. J. Biomed. Opt..

[38] Jacques S.L. (2013). Optical properties of biological tissues: A review. Phys. Med. Biol..

[39] Sawosz P., Wojtkiewicz S., Kacprzak M., Weigl W., Borowska-Solonynko A., Krajewski P., Liebert A. (2016). Human skull translucency: Post mortem studies. Biomed. Opt. Express.

[40] Axer M., Amunts K., Grässel D., Palm C., Dammers J., Axer H., Zilles K. (2011). A novel approach to the human connectome: Ultra-high resolution mapping of fiber tracts in the brain. NeuroImage.

[41] Wan P., Zhu J., Xu J., Li Y., Yu T., Zhu D. (2018). Evaluation of seven optical clearing methods in mouse brain. Neurophotonics.

[42] Oliveira L.M., Tuchin V.V. (2019). The Optical Clearing Method—A New Tool for Clinical Practice and Biomedical Engineering.

[43] Biswas T., Luu T. (2009). In vivo MR Measurement of Refractive Index Relative Water Content and T2 Relaxation time of Various Brain lesions with Clinical Application to Discriminate Brain Lesions. Internet J. Radiol..

[44] Sydoruk O., Zhernovaya O.S., Tuchin V.V., Douplik A. (2012). Refractive index of solutions of human hemoglobin from the near-infrared to the ultraviolet range: Kramers-Kronig analysis. J. Biomed. Opt..

[45] Bashkatov A.N., Genina E.A., Kozintseva M.D., Kochubei V.I., Gorodkov S.Y., Tuchin V.V. (2016). Optical properties of peritoneal biological tissues in the spectral range of 350–2500 nm. Opt. Spectrosc..

[46] Biswas T.K. (2009). A process for tissue characterization of brain and brain tumor obtained from MR images by using deviations of refractive indices. J. Indian Pat. Inf. Retr. Syst..

[47] Deng Z., Wang J., Ye Q., Sun T., Zhou W.Y., Mei J., Tian J. (2016). Determination of continuous complex refractive index dispersion of biotissue based on internal reflection. J. Biomed. Opt..

